# Dynamic Stability and Risk of Tripping during the Timed Up and Go Test in Hemiparetic and Healthy Subjects

**DOI:** 10.1371/journal.pone.0140317

**Published:** 2015-10-15

**Authors:** Céline Bonnyaud, Didier Pradon, Djamel Bensmail, Nicolas Roche

**Affiliations:** 1 Inserm Unit 1179, Team 3: Technologies and Innovative Therapies Applied to Neuromuscular diseases, UVSQ, CIC 1429, APHP Laboratoire d’analyse du mouvement, Service de physiologie et d’exploration fonctionnelle, Hôpital Raymond Poincaré, 92380, Garches, France; 2 Inserm Unit 1179, Team 3: Technologies and Innovative Therapies Applied to Neuromuscular diseases, UVSQ, CIC 1429, APHP Service de médecine physique et réadaptation, Hôpital Raymond Poincaré, 92380, Garches, France; INSERM U894, FRANCE

## Abstract

**Background:**

The Timed Up and Go (TUG) test is often used to estimate risk of falls. Foot clearance and displacement of the center of mass (COM), which are related to risk of tripping and dynamic stability have never been evaluated during the TUG. Accurate assessment of these parameters using instrumented measurements would provide a comprehensive assessment of risk of falls in hemiparetic patients. The aims of this study were to analyze correlations between TUG performance time and displacement of the COM and foot clearance in patients with stroke-related hemiparesis and healthy subjects during the walking and turning sub-tasks of the TUG and to compare these parameters between fallers and non-fallers.

**Methods:**

29 hemiparetic patients and 25 healthy subjects underwent three-dimensional gait analysis during the TUG test. COM and foot clearance were analyzed during the walking and turning sub-tasks of the TUG.

**Results:**

Lateral displacement of the COM was greater and faster during the walking sub-tasks and vertical displacement of the COM was greater during the turn in the patients compared to the healthy subjects (respectively p<0.01 and p<0.05). Paretic foot clearance was greater during walking and displacement of the COM was slower during the turn in the patients (p<0.01). COM displacement and velocity during the turn were correlated with TUG performance in the patients, however, vertical COM displacement was not. These correlations were significant in the healthy subjects. There were no differences between COM parameters or foot clearance in fallers and non-fallers.

**Discussion and Conclusion:**

Hemiparetic patients are less stable than healthy subjects, but compensate with a cautious gait to avoid tripping. Instrumented analysis of the TUG test appears relevant for the assessment of dynamic stability in hemiparetic patients, providing more information than straight-line gait.

## Introduction

Stroke related impairments such as sensorimotor dysfunction affect balance and gait, increasing the risk of falls. Falls are costly to the health system and are therefore an issue of public health [1],[2]. Two thirds of falls reported by stroke patients living at home occur during gait-related activities [1],[3]. Direction changes and turns are particularly hazardous [1],[4]. The majority of patients relate falls to intrinsic factors such as impaired balance and foot dragging [4],[2]. Dynamic stability (the ability to move without loss of balance) during gait related activities (walking, turning etc.) is essential for autonomy and should be assessed. Two biomechanical parameters, the control of the center of mass (COM) and foot clearance, are considered pertinent for the evaluation of dynamic stability and risk of tripping [4],[5],[6],[7].

The Timed Up and Go test (TUG) assesses gait related activities which involve dynamic stability. It involves rising from a chair, walking 3 meters, turning, walking back and sitting down again. The task thus corresponds to activities regularly encountered in daily life [8]. This test is widely used and is validated in stroke patients [9]. Performance is measured as the time to carry out the test. It has been shown to be useful to identify fallers and non-fallers among older subjects and stroke patients [10],[11],[12] however recent evidence suggests that its sensitivity is low and its ability to predict falls is limited [11],[13],[14]. Balance capacity is the main predictor of falls and the relevance of the TUG for the assessment of balance and mobility has been well demonstrated [12],[13]. However, performance time may not be a relevant criterion for the accurate assessment of dynamic stability. Zampieri et al (2010) carried out an instrumented evaluation of the TUG in people with Parkinson’s disease [15]. They found no difference in TUG performance time between patients and healthy subjects but highlighted differences in spatio-temporal parameters during the walking and turning sub-tasks using accelerometers. This suggests that the evaluation of biomechanical parameters is particularly pertinent to quantify dynamic stability and therefore to identify the main parameters related to the risk of falls in each patient.

The displacement and velocity of the COM have been shown to be pertinent for the assessment of dynamic stability during locomotion [6], [16]. The amplitude and lateral velocity of the COM is increased during obstacle crossing in elderly individuals with loss of balance capacity [17] as well as in subjects with brain injury [18],[19]. Vertical COM displacement is also increased during gait in hemiparetic patients compared with healthy subjects [20]. Thus assessment of COM displacement appears to be a useful parameter for the evaluation of dynamic stability in stroke patients.

Another useful parameter is minimum foot clearance (MFC), defined as the minimum vertical distance between the lowest part of the foot of the swing leg and the walking surface during the swing phase of the gait cycle [5]. Foot clearance is the result of shortening of the lower limb due to a combination of hip, knee and ankle joint flexion. Foot trajectory is the primary mode of error correction to allow stability during gait. Its analysis thus provides information regarding dynamic stability while walking [21]. Patients with chronic stroke report lack of foot clearance as being a cause of falls [4],[7]. However, Little et al (2014) recently investigated foot clearance of the paretic limb in 16 stroke patients and found that it was increased compared to healthy subjects [22]. Thus the assessment of both COM displacement and foot clearance should provide an accurate assessment of dynamic stability, helping to increase understanding of the main biomechanical determinants of TUG test performance and to define which parameters are particularly related to the risk of falls in patients with stroke [2],[4],[23]. Such a comprehensive assessment may identify potential fallers who could then be targeted in falls prevention programs. Moreover, recent guidelines highlighted the importance of objectively assessing dynamic stability in hemiparetic patients during gait and activities of daily living [21],[24].

The aims of this study were (i) to analyse the vertical and mediolateral displacement of the COM and foot clearance in hemiparetic patients, and to compare them with healthy subjects during the walking and turning sub-tasks of the TUG test; (ii) to evaluate the relationship between COM parameters and foot clearance and TUG performance during the same TUG sub-tasks; and (iii) to compare COM and foot clearance parameters between fallers and non-fallers with hemiparesis. We hypothesized that the lateral and vertical displacement of the COM would be greater and faster, and that MFC would be greater in patients with stroke than in healthy subjects. We also hypothesized that COM parameters and MFC would be positively correlated with TUG sub-task performance time, and that COM displacement would be greater and MFC smaller in fallers with hemiparesis compared with non-fallers with hemiparesis.

## Methods

### Subjects

Twenty nine patients with chronic hemiparesis (mean age 54.2±12.2 years) and twenty five healthy subjects (mean age 51.6±8.7 years) participated in this study. This number of subjects was sufficient for a statistical power of 95%, based on the computation of the effect size and statistical power using previous data in the literature [9][25], and validated a posteriori with the results of the present study [26]. Table 1 presents the characteristics of the participants. All the patients were able to walk without assistance, the median New Functional Ambulation Classification index was 7 (min 6 and max 8), the median lower limb strength score on the Medical Research Council scale was 4 (min 2 and max 5), the median Berg Balance Scale score was 51 (min 45 and max 54) and the mean TUG time was 19.3±4.2sec. The inclusion criteria for the patients were: age over 18 years, hemiparesis due to stroke, ability to carry out several TUG tests without the use of an assistive device and medically stable enough for participation in the protocol. Patients were excluded if they had other neurological, orthopedic or medical disorders that might interfere with the test. Falls were defined as any event that led to an unplanned, unexpected contact with a supporting surface [10]. According to this definition, 14 patients fell during the 3 months prior to inclusion. Two of these patients were not considered as fallers in this study since they did not fall during gait (one fell in the bathtub and the other entering a car). The fallers group therefore consisted of 12 patients, and the non-fallers group of 17 patients. The healthy subjects had no neurological or orthopedic impairments. All patients and healthy subjects gave their written informed consent in accordance with the ethical codes of the World Medical Association and the guidelines of our local ethics committee who approved the study (Comité de protection des personnes Ile de France XI, Ref 13005. CNIL, Ref DR-2013-283).

**Table 1:** Subject characteristics.

	Age (years)	Height (m)	Weight (kg)	Gender (m/f)	Time since stroke (years)	Hemiparetic side
Hemiparetic patients	54.2±12.2	1.68±0.09	73.2±16.2	18m/ 11f	7.9±5.7	12 right / 17 left
Healthy subjects	51.6±8.7	1.67±0.1	65.6±14.7	11m/ 14f	-	-

### Experimental procedure

Three-dimensional (3D) kinematic data were recorded while subjects performed the TUG test. Thirty-four markers placed on anatomical landmarks according to the Helen Hayes marker set [27],[28],[29] were tracked by an optoelectronic motion capture system (sampling frequency 100 Hz, Motion Analysis Corporation, Santa Rosa, CA, USA). The same person positioned all the markers to ensure good reliability. The greater trochanter and the anterior superior iliac spine were added to improve the reconstruction of the trajectories of joint coordinate systems. This marker set allowed the creation of a 12-segment rigid-link model of the body, using Dempster's anthropometric table which is routinely used in gait analysis [30],[31].

All participants performed 3 TUG tests which involved rising from a stool, walking 3m, turning around a cone and returning to sit, at their own comfortable speed. The original TUG test involves a standard chair with armrests and does not specify the subject’s position or the direction of the turn [8]. However to ensure the reliability of the results, the conditions were standardized [32][33][34]. Subjects sat on a stool set to 100% of the distance from the head of the fibula to the floor [35], their knees were flexed to 100°, feet were placed symmetrically and arms were held out from the body [32],[33],[36],[37].

Marker trajectories were filtered using a low-pass Butterworth filter with a cut off frequency of 6 Hz [38]. The phases of the gait cycle were defined according to Perry [39] and were determined using the Open-source Biomechanical Tool Kit package for MATLAB [40]. This tool was also used to determine the 3 sub-phases of the TUG test (walking toward the cone (GO); turning (Turn), return to the stool (Return)), according to previous studies [37],[41],[42]. The data were then exported to Matlab (R14, The MathWorks Inc., Natick, MA, USA) for calculation of the biomechanical parameters.

The two walking sub-tasks (Go and Return) and the turning sub-task (Turn) of the TUG were analyzed. The sit-to-stand and stand-to-sit sub-tasks were not considered since they have already been largely evaluated in patients with stroke [35], [32]. The time taken to perform each sub-task was measured.

Markers and estimated joint centers were used to calculate the center of mass (COM) of each individual body segment [43].

Whole body COM position data were then calculated with the following Eq 1:

$$COMx=\frac{m_{1}\;x_{1\;}+\;m_{2}\;x_{2\;}+\;{\ldots ..+\;m}_{i}\;x_{i}\;}{M}=\frac{1}{M}\sum_{i=1}^{N}m_{i}\;x_{i}$$

where M = whole body mass

mi = mass of the ith segment = (whole body mass) x (mass fraction for ith segment from the anthropometrics.dat file)

xi = the x-coordinate of the center of mass for the ith segment with respect to the calibration origin

N = the number of body segments

COM movements were analyzed in the subject reference frame with respect to the line of gait, considered as the trajectory of the sacral marker.

The amplitude and velocity of COM displacement in the mediolateral (ML) and vertical (Vert) directions were analyzed. The ML-COM displacement was the distance between the most leftward and rightward positions of the COM and the Vert-COM displacement was the distance between the highest and lowest positions of the COM. The maximum velocity of COM displacement was also calculated in the mediolateral (ML-V) and vertical (Vert-V) directions.

MFC was calculated by subtracting the height of the toe marker (between the second and the third toe) during mid-stance from the minimum height of the toe marker during mid-swing, for each gait cycle and for both limbs, since reduced MFC at this instant indicates an increased likelihood of tripping [44],[45],[46].

### Statistical analysis

Descriptive statistics including means and standard deviations were calculated for each parameter and each sub-task of the TUG (Go, Turn, Return). The data were normally distributed according to the Shapiro Wilk test. To compare hemiparetic patients and healthy subjects, intergroup analysis, independent Student t tests were used for each TUG sub-task. To compare sub-tasks (Go, Turn and Return) in each group (intragroup analysis) repeated measures ANOVA were carried out. Tukey post hoc tests were then performed on significant comparisons. Correlations between TUG performance and COM and MFC parameters were tested with Pearson’s correlations for both the hemiparetic patients and healthy subjects. The *r* values were interpreted according to Domholdt [47]. To compare COM and MFC parameters between fallers and non-fallers with hemiparesis, independent Student t tests were used. All significance levels were set at p < 0.05.

## Results

Results of the TUG performance time and COM and MFC parameters for each sub-task and both groups are presented in Table 2.

**Table 2:** TUG performance, COM parameters and foot clearance in hemiparetic patients and healthy subjects during Go, Turn and Return sub-tasks.

	Hemiparetic patients			Healthy subjects		
	Go	Turn	Return	Go	Turn	Return
TUG performance time (sec)	4.56 (1.01)	3.16 (0.84)	3.81 (0.91)†	2,44 (0,28)*	1,41 (0,25)*	2,28 (0,45)* †
MFC on paretic side (cm)	2,84 (1,18)	3,71 (1,54)	3,72 (1,32)†	1,80 (0,75)*	3,21 (1,55)	2,52 (0,75)* †
MFC on non-paretic side (cm)	1,92 (1,11)	2,26 (1,00)	2,75 (1,00)†	1,98 (0,85)	3,00 (1,02)*	2,72 (0,60)†
ML-COM	8,91 (1,82)	19,02 (4,39)	9,19 (2,04)†	6,71 (1,60)*	25,36 (4,10)*	8,61 (2,01)†
ML-V	23,04 (4,38)	30,15 (5,91)	22,61 (4,56)†	16,97 (2,34)*	57,50 (10,56)*	16,51 (2,61)* †
Vert-COM	4,48 (1,07)	3,58 (0,78)	4,56 (0,99)†	4,24 (0,68)	3,14 (0,74)*	4,14 (0,67)†
Vert-V	18,93 (4,72)	15,50 (3,61)	20,48 (4,32)†	20,92 (4,42)	16,15 (4,35)	20,83 (3,84)†

* significant difference between hemiparetic patients and healthy subjects for the corresponding sub-task of the TUG p<0,05

† significant difference between Go, Turn and Return p<0.05

### Differences between hemiparetic patients and healthy subjects: inter-group analysis

#### COM

**Fig 1:**
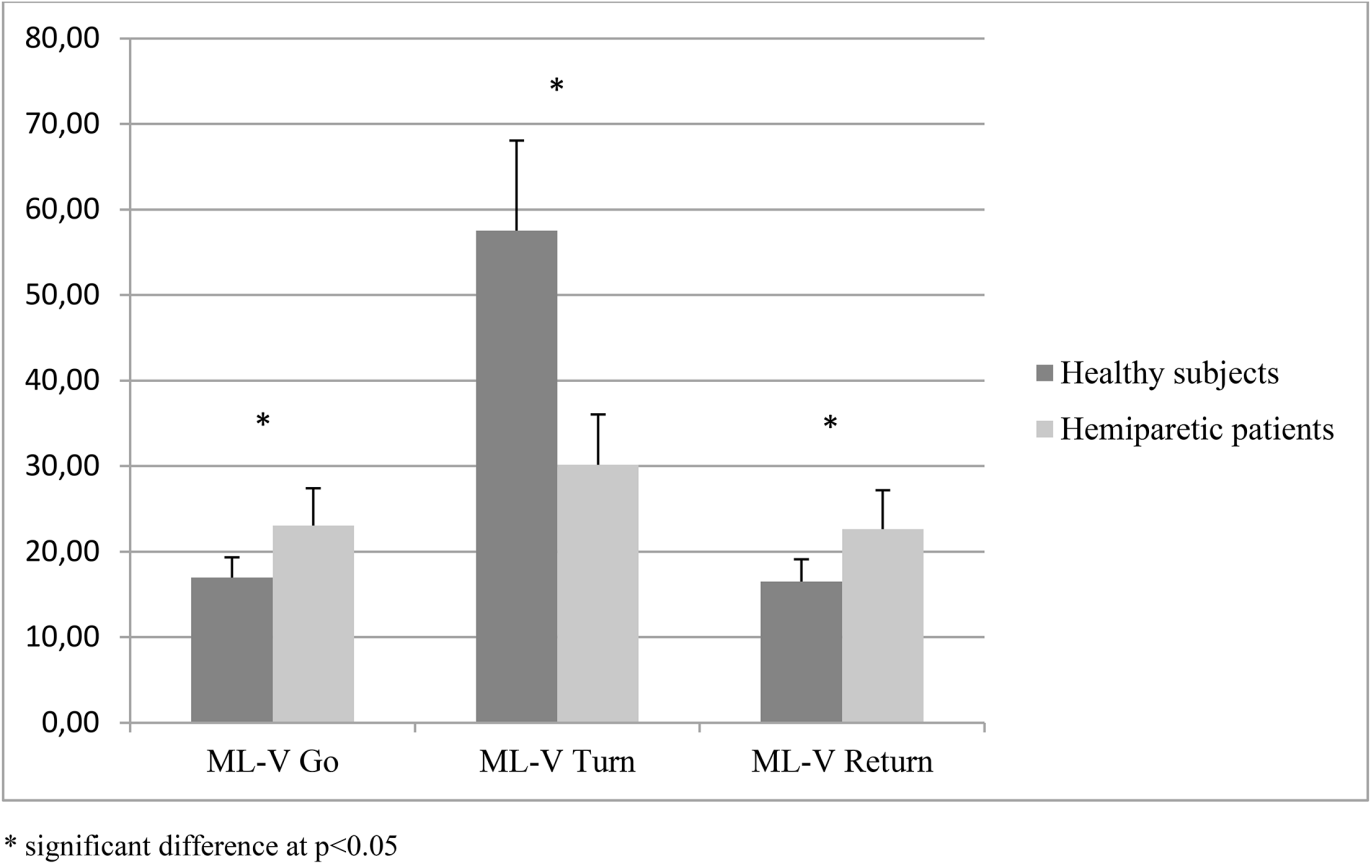
Medio-lateral COM velocity.

Compared to healthy subjects, ML-COM amplitude was significantly greater for hemiparetic patients during Go, significantly smaller during Turn and was not different during Return. ML-V was higher for hemiparetic patients during Go and Return, and smaller during Turn (Fig 1). Vert-COM was significantly greater for hemiparetic patients during Turn and was not different during the Go and Return sub-tasks. There were no differences between groups for Vert-V.

#### MFC

**Fig 2:**
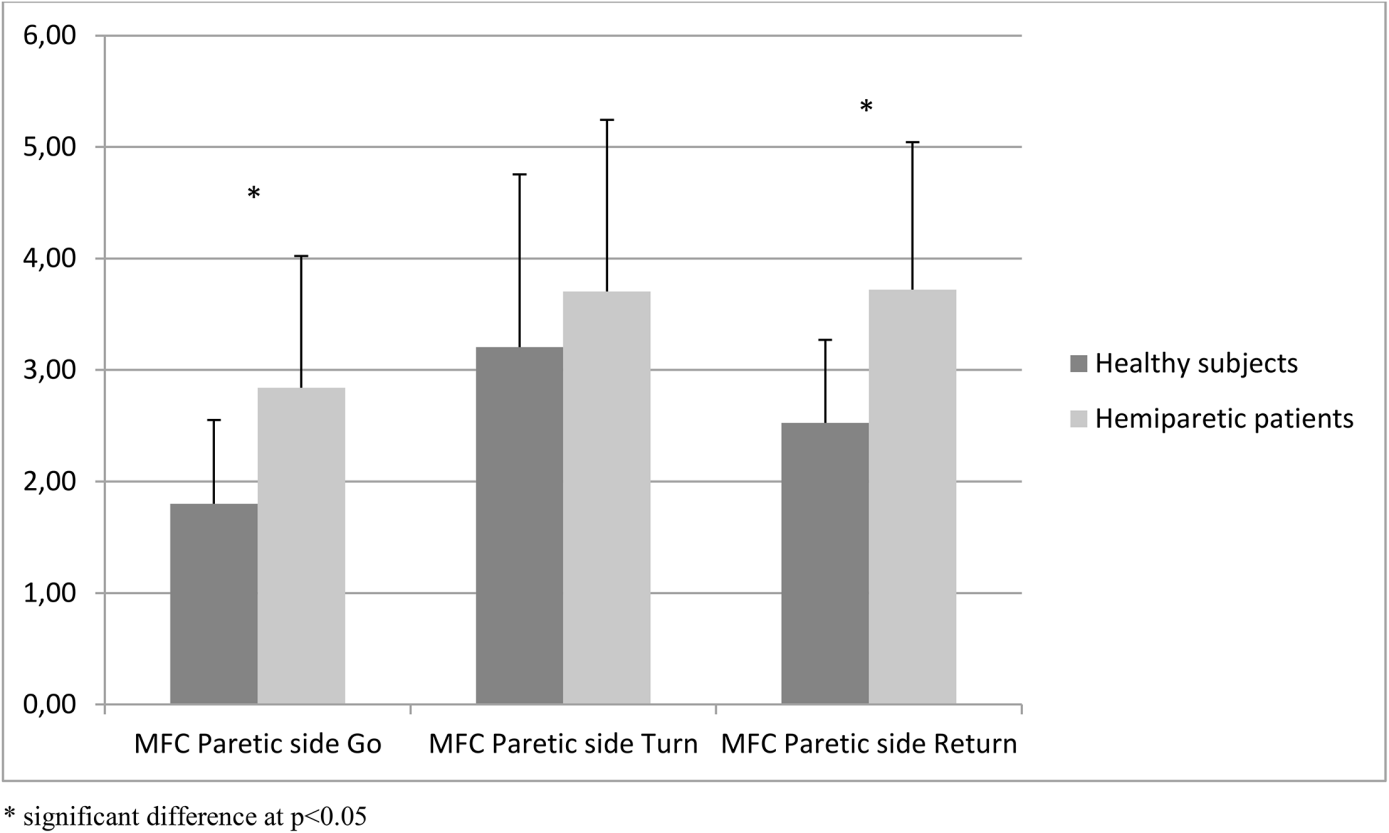
Minimum foot clearance on paretic side.

MFC on the paretic side was significantly greater for hemiparetic patients during Go and Return sub-tasks when compared to healthy subjects but was not different during Turn (Fig 2). MFC on the non-paretic side was significantly smaller for hemiparetic patients during Turn when compared to healthy subjects but was not different during the Go and Return sub-tasks.

### Difference between sub-tasks of the TUG for each group: intra-group analysis

#### COM

There were significant differences between sub-tasks for both groups for ML-COM, ML-V, Vert-COM and Vert-V, except for Vert-COM between Go and Return for both groups.

#### MFC

There were significant differences between sub-tasks for both groups for MFC, except between Turn and Return for the paretic side in the hemiparetic patients.

### Correlations between TUG performance and, COM and MFC parameters (Table 3)

**Fig 3:**
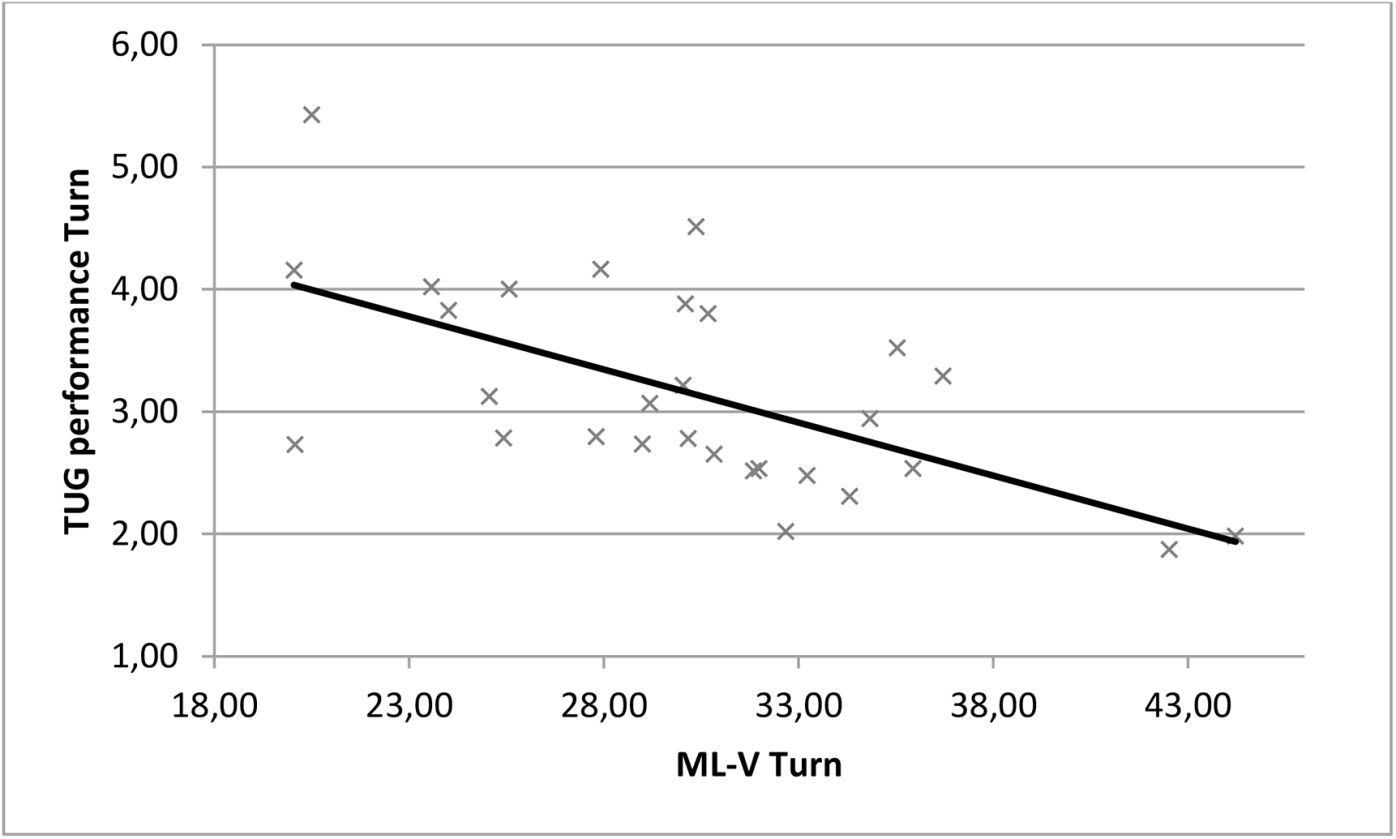
Correlation between ML COM velocity and TUG performance time during the turning sub-task in hemiparetic patients.

There were significant correlations between TUG performance time and ML-COM (r = -0.59, p = 0.001) and ML-V (r = -0.61, p = 0.0001, Fig 3) for the Turn in the hemiparetic patients but not for the healthy subjects. There was also a significant correlation between TUG performance time and Vert-V for the Return in both groups. There were no significant correlations between the other parameters and other sub-tasks. There were significant correlations between TUG performance time and Vert-COM and Vert-V for Go and Return in the healthy subjects but not the hemiparetic patients.

**Table 3:** Correlations between TUG performance time, and COM and MFC parameters for hemiparetic patients and healthy subjects.

	Hemiparetic patients			Healthy subjects		
	Go	Turn	Return	Go	Turn	Return
MFC on paretic side (cm)	R = 0.31	R = -0.12	R = 0.29	R = -0.13	R = -0.09	R = -0.13
MFC on non-paretic side (cm)	R = 0.33	R = 0.22	R = 0.13	R = -0.01	R = -0.09	R = -0.17
ML-COM	R = 0.31	R = -0.59*	R = 0.25	R = -0.09	R = -0.38	R = -0.03
ML-V	R = 0.13	R = -0.61*	R = 0.008	R = 0.09	R = -0.39	R = -0.38
Vert-COM	R = -0.12	R = -0.04	R = -0.06	R = -0.47*	R = -0.04	R = -0.55*
Vert-V	R = -0.19	R = -0.30	R = -0.43*	R = -0.71*	R = -0.27	R = -0.64*

* significant correlation between TUG performance and the corresponding parameter at p<0,05

### Difference between fallers and non-fallers for COM and MFC parameters

12 hemiparetic patients constituted the fallers group and 17 patients constituted the non-fallers group. No differences were found between fallers and non-fallers for total TUG time (respectively 18.61±2.78sec and 19.76±5.04) or time to perform the Go (respectively 4.42±0.94sec and 4.66±1.07), Turn (respectively 2.97±0.75sec and 3.29±0.90) and Return (respectively 3.55±0.79sec and 4.00±0.97) TUG sub-tasks. Vert-V was higher for the fallers compared to the non-fallers during the Turn (respectively 17.1±3.7cm/sec and 14.3±3.1cm/s, p = 0.04) but did not differ between these groups during Go and Return. There were no differences between fallers and non-fallers during any TUG sub-task for ML-COM and ML-V. There were no differences in MFC for either foot (paretic and non-paretic) between fallers and non-fallers.

## Discussion

This is the first study to objectively and accurately assess dynamic stability and foot clearance during a goal-directed walking task involving turning in hemiparetic patients. This assessment is in line with recent recommendations regarding falls risk [21],[24] since impaired balance while walking and potential foot dragging increase the risk of falls [2],[4],[23]. The aims of this study were (i) to compare vertical and mediolateral displacements of the COM and foot clearance between hemiparetic patients and healthy subjects during the walking and turning sub-tasks of the TUG test; (ii) to analyze the relationship between COM and clearance parameters and TUG performance during these TUG sub-tasks; and (iii) to compare COM and clearance parameters between faller and non-faller patients with hemiparesis. The results showed that the majority of parameters studied differed between hemiparetic patients and healthy subjects. The values of most parameters were greater during the walking sub-tasks and lower during the Turn in the hemiparetic patients compared with the healthy subjects, except for vertical COM displacement.

We hypothesized that lateral and vertical COM displacement would be greater and faster in the hemiparetic patients during the walking and turning sub-tasks of the TUG. This hypothesis was confirmed for the ML direction during the walking sub-tasks (except ML-COM on Return). This is in accordance with previous studies in the elderly and subjects with brain injury during gait and obstacle crossing [17],[18],[19],[48]. This result reflects greater instability in hemiparetic patients compared to healthy subjects when walking towards a goal and preparing to turn. This might be explained by the fact that healthy subjects minimized lateral displacements in order to maximize forward body transfer [49].

The motor behavior during the turning sub-task was interesting. This task involved turning around a cone, consisting essentially of a rotation of the body toward the new direction with a lateral translation of the COM [50]. Displacement of the COM in the ML direction was smaller and slower during the Turn in the patients, reflecting a less efficient movement than the healthy subjects. Such adaptations have already been evoked by Patla et al (1999) and Vallis et al (2004) who suggested that healthy subjects reduce motion of the COM when directional changes are required [51],[52]. Similarly Hollands et al (2001) showed that when healthy subjects turn, there is a lateral displacement of the COM which reflecting translation of the body in the direction of the turn [50]. It could be hypothesized that hemiparetic patients slow lateral COM movement to increase stability. Recently Hurt et al (2015) found that lateral COM velocity was greater in young adults performing a lateral step during forward walking compared to older adults [53]. The authors suggested that the younger subjects favored maneuverability whereas the older subjects favored stability. The findings of the present study during the turn sub-task are in accordance with these results. Moreover, ML displacement and velocity of the COM were greater in both the patients and the healthy subjects during turning compared to the walking sub-tasks. This is not surprising since turning induces more movement in ML direction relative to walking forward.

Surprisingly, there were no differences between the groups for vertical displacement and velocity of the COM during the walking sub-tasks. This contrasts with the results of Detrembleur et al (2003) which showed increased vertical displacement of the COM during walking in hemiparetic patients compared to healthy subjects [20]. Our result is nevertheless in accordance with Chou et al (2004) who found no differences in vertical COM between subjects with brain injury and healthy subjects during obstacle crossing [18]. The differences between these results may be related to differences in the tasks evaluated: goal-oriented gait in the present study, straight-line walking in the study by Detrembleur et al (2003) and obstacle crossing in the study by Chou et al (2004).

During the turning sub-task, we found greater vertical displacement of the COM in the patients compared to healthy subjects (no difference for velocity). This likely reflects greater instability when performing movements in the ML plane since stability requires online control of COM displacement. Healthy subjects may control vertical COM displacement in order to ensure the efficiency of lateral movements during a turn. Turning requires altering the spatial reference to focus on the ML direction in contrast with walking forward. This type of task thus seems to affect vertical COM movements.

We hypothesized that COM movements would be positively correlated with the time to perform the sub-tasks of the TUG. The results showed that ML COM displacement and velocity were significantly negatively correlated (moderate correlations according to Domholdt) with TUG performance time during the turning sub-task in the hemiparetic patients. This reinforces our previous argument that efficient turning requires sufficient ML displacement [50],[51],[53]. Vertical COM movements were not correlated with TUG performance in the hemiparetic patients but were negatively correlated with performance time for the walking sub-tasks in the healthy subjects. Positive relationships have been found between increased gait velocity and increased vertical COM movements in healthy subjects, which is in accordance with our results [49],[54]. The relationship between ML COM parameters and performance time in the hemiparetic patients suggests that ML COM parameters are more relevant than vertical COM parameters for the assessment of dynamic stability and to explain the performance of hemiparetic patients during the TUG test.

We expected that hemiparetic patients would exhibit greater MFC in comparison with healthy subjects. Our results partly confirmed this hypothesis, showing that MFC was greater on the paretic side during the walking sub-tasks but not during turning. These results are in accordance with those of Little et al (2014) who also found an increase in foot clearance during walking in hemiparetic patients compared to healthy subjects [22]. They assessed 16 individuals with stroke during over-ground walking at self-selected speed and 9 non-disabled control subjects, and found MFCs of respectively 3.25±0.34cm and 1.48±0.69cm. Winter (1992) reported a MFC of around 1.29cm for healthy subjects [44]. In the present study, we found higher MFCs during the walking sub-tasks for both groups (see Table 2). This difference could be due to the type of the task assessed, walking in anticipation of turning may be more complex than walking in a straight line as in the studies by Little et al and Winter. It was also interesting to note that, although during the walking sub-tasks there were no differences between non-paretic MFC and healthy subject MFC, non-paretic MFC was lower during the turn. This was not the case for paretic MFC. Turning while walking is a complex task requiring more control than straight walking to avoid tripping. This may explain the greater MFC in the healthy subjects during turning compared to the walking sub-tasks. Similarly, paretic MFC was increased between Go and Turn in the hemiparetic patients and remained increased for Return on the paretic side, or increased more for Return on the non-paretic side. We could hypothesize that the higher MFC during walking in hemiparetic patients and during turning in both groups reflect adaptations to potentially complex situations requiring greater control. This is corroborated by other studies. Heasley et al (2004) found a significant increase in MFC when healthy subjects stepped up with blurred vision compared with clear vision, suggesting that the safety margin is increased in uncertain conditions [55]. MFC is also increased when walking over rocky ground compared with smooth [56]. These results all suggest that MFC is increased in complex or uncertain conditions to reduce the risk of tripping [55],[56],[57] whatever the population studied. This parameter thus provides information regarding gait adaptations to complex conditions, but is not correlated with TUG performance time in hemiparetic patients.

We also hypothesized that COM movements would be greater and MFC smaller in fallers with hemiparesis than non-fallers. The TUG test is considered to be useful to identify fallers and non-fallers among older subjects and stroke patients [10],[11],[12], however our results showed that the time taken to carry out each sub-task of the total TUG did not differ between fallers and non-fallers. The only parameter which differed between fallers and non-fallers was the vertical velocity of the COM during the turning sub-task. This parameter is related to dynamic stability and can thus distinguish fallers from non-fallers during a complex locomotor task. MFC (related to the risk of tripping) and ML COM displacement did not differ between fallers and non-fallers. This is in accordance with a recent review in elderly subjects stating that MFC does not generally differ between fallers and non-fallers [7].

### Limits

Displacement of the COM should be interpreted with caution. This parameter depends on balance capacity; previous studies found greater displacement of the COM in subjects with impaired balance [18],[19],[20]. However, it also depends on gait speed [49],[54]. Orendurff et al (2004) and Staszkiewicz et al (2010) showed that vertical COM displacement increased and lateral COM displacement decreased with increasing gait velocity in healthy subjects [49],[54]. In the present study, the gait speed of the patients was lower than the healthy subjects, thus, according to the literature, COM displacement should have been smaller in the vertical direction and greater in the lateral direction compared to healthy subjects for the walking sub-tasks. However COM parameters were either greater in the hemiparetic patients or not different between groups, thus we can be confident with our previous interpretation. Hamacher et al (2011) reviewed studies of gait stability in elderly subjects and suggested that the analysis of variability is the most pertinent assessment to differentiate fallers from non-fallers [21]. Variability of MFC is greater in older fallers compared to older non-fallers [7]. We could not analyze this parameter because of the small number of gait cycles involved in the TUG test. However, it might be interesting to carry out an analysis of variability in further studies with a large number of trials and a large number of gait cycles.

## Conclusion

This study presents an innovative approach for the assessment of dynamic stability and risk of tripping during gait-related activities of daily living in hemiparetic patients, as has been recommended [21],[24]. The results suggest that the analysis of ML COM parameters is relevant for the assessment of dynamic stability and to explain TUG performance in hemiparetic patients. ML COM velocity decreased during turning, reflecting cautious gait. It increased during oriented-gait, reflecting instability. Vertical COM velocity during the turn distinguished fallers from non-fallers. Turning appears to be a relevant locomotor task to analyze dynamic stability and risk of falling. MFC reflected adaptations during goal-oriented gait in the hemiparetic patients but was not related to performance time. The instrumented analysis of gait-related activities of daily living thus has important clinical applications. Accurate analysis of COM displacements during oriented gait and turning tasks of the TUG in hemiparetic patients is useful to understand instability and risk of falling. Further studies assessing the effects of rehabilitation programs on the control of dynamic stability and risk of tripping in hemiparetic patients would be useful.
